# Can Multiple Lifestyle Behaviours Be Improved in People with Familial Hypercholesterolemia? Results of a Parallel Randomised Controlled Trial

**DOI:** 10.1371/journal.pone.0050032

**Published:** 2012-12-12

**Authors:** Karen Broekhuizen, Mireille N. M. van Poppel, Lando L. Koppes, Iris Kindt, Johannes Brug, Willem van Mechelen

**Affiliations:** 1 Department of Public and Occupational Health, EMGO+ Institute for Health and Care Research, VU University Medical Centre, Amsterdam, The Netherlands; 2 Division of Work and Employment, TNO, Hoofddorp, The Netherlands; 3 Foundation for the Identification of Persons with Inherited Hypercholesterolemia, Amsterdam, The Netherlands; 4 EMGO+ Institute for Health and Care Research, VU University Medical Centre, Amsterdam, The Netherlands; University of Ottawa, Canada

## Abstract

**Objective:**

To evaluate the efficacy of an individualised tailored lifestyle intervention on physical activity, dietary intake, smoking and compliance to statin therapy in people with Familial Hypercholesterolemia (FH).

**Methods:**

Adults with FH (n = 340) were randomly assigned to a usual care control group or an intervention group. The intervention consisted of web-based tailored lifestyle advice and face-to-face counselling. Physical activity, fat, fruit and vegetable intake, smoking and compliance to statin therapy were self-reported at baseline and after 12 months. Regression analyses were conducted to examine between-group differences. Intervention reach, dose and fidelity were assessed.

**Results:**

In both groups, non-significant improvements in all lifestyle behaviours were found. Post-hoc analyses showed a significant decrease in saturated fat intake among women in the intervention group (β = −1.03; CI −1.98/−0.03). In the intervention group, 95% received a log on account, of which 49% logged on and completed one module. Nearly all participants received face-to-face counselling and on average, 4.2 telephone booster calls. Intervention fidelity was low.

**Conclusions:**

Individually tailored feedback is not superior to no intervention regarding changes in multiple lifestyle behaviours in people with FH. A higher received dose of computer-tailored interventions should be achieved by uplifting the website and reducing the burden of screening questionnaires. Counsellor training should be more extensive.

**Trial Registration:**

Dutch Trial Register NTR1899

## Introduction

Familial hypercholesterolemia (FH) is an autosomal dominant disorder of the lipoprotein metabolism. Due to a defect of the low density lipoprotein (LDL) receptor gene, plasma concentrations of LDL cholesterol (LDL-C) are elevated [1]. In most Western countries, approximately one in 500 people is affected with FH [2]. Elevated serum LDL-C and therefore FH is associated with an elevated risk of premature cardiovascular disease (CVD) [3], which is the disease with the highest burden in disability adjusted life years in the Netherlands [4]. If elevated LDL-C is not diagnosed and treated, the cumulative risk of developing coronary artery disease by the age of 60 years is over 60% for men, and over 30% for women [5].

Yet, research has mainly been focused on the effectiveness of pharmaceutical therapy, whereas achieving (additional) improvement by lifestyle change has hardly been investigated in people with FH. Large primary and secondary prevention trials with statins have clearly demonstrated the benefit of reducing LDL-C in subjects with high LDL-C [6], [7]. Also, Versmissen and colleagues showed an overall risk reduction in a large cohort (n = 2146) of people with FH that used statins [8]. However, lifestyle factors also appear to play an important role in moderating the course of FH [9], [10]. The EUROASPIRE III survey, conducted in 2006–2007 in 22 European countries, showed a high prevalence of unhealthy lifestyles among CVD patients treated by cardiologists, and moreover, use of medication was often inadequate to achieve treatment goals [11]. Overall, two main strategies are of importance to optimally reduce CVD risk among people with FH: 1) Improvement of compliance to statin therapy, and 2) Improvement of CVD-risk-related lifestyle.

A healthy lifestyle is mentioned as an aspect of the treatment of FH with many benefits beyond LDL-C-lowering drugs [12]. In the most recent European guidelines on cardiovascular disease prevention [13], lifestyle modification is recommended for individuals at high risk for CVD. Results of primary prevention trials in high-risk persons and secondary prevention trials in CVD patients both show that substantial reductions in the CVD risk can be obtained through lifestyle changes [14]–[15]. For example, the INTERHEART study showed that eating fruit and vegetables daily, being physically active regularly and avoiding smoking were effective in reducing the risk of a myocardial infarction by 80% [16]. Particularly, interpersonal and tailored interventions matching an individual's specific needs and preferences have shown promising results within a range of lifestyle behaviours [17], [18].

There is a lack of evidence-based interventions that incorporate a comprehensive approach to optimise treatment goals of people with FH in the Netherlands, as well as elsewhere. We assume that lifestyle improvements can positively change biological CVD risk indicators, and that this would eventually lead to a reduction of the CVD risk. In the PRO-FIT project, we developed an individually tailored lifestyle intervention aimed at a CVD risk reduction in individuals with FH. At first, we investigated the efficacy of the intervention on biological CVD risk indicators: lipids (LDL-C, HDL-C, TC and triglycerides), systolic blood pressure, glucose, body mass index (BMI) and waist circumference [19]. In this paper, we report the efficacy on smoking, physical activity, dietary intake and compliance to statin therapy.

## Materials and Methods

### Design and participants

The protocol for this trial and supporting CONSORT checklist are available as supporting information; see Checklist S1 and Protocol S1. A parallel randomised controlled trial was conducted with measurements at baseline and at 12 months post-baseline. Participants diagnosed with FH through DNA analyses from January 1^st^ 2007 to April 15^th^ 2009, aged from 18 to 70 years and with a LDL-level>75^th^ percentile (age and gender specific) were recruited from the national cascade screening program of the Foundation for the Identification of Persons with Inherited Hypercholesterolemia (StOEH) [20]. Access to internet, sufficient fluency in Dutch and residency <150 km radius from Amsterdam were additional eligibility criteria. Invitation brochures were send by a research assistant to 986 people during six months (from February 1^st^ 2009 until August 1^st^ 2009) and resulted in 340 participants (34%), of whom 336 (99%) completed the baseline questionnaire, and 318 (94%) completed the baseline and follow-up questionnaire. The follow-up period of 12 months lasted until August 2010. Details on recruitment and participant flow can be found in figure S1. Details on power calculation can be found elsewhere [21].

The content of this paper was guided by the recommendations for reporting randomised controlled trials of the CONSORT (Consolidated Standards of Reporting Trials) statement [22]. The PRO-FIT project was approved by the Medical Ethics Committee of the VU University Medical Centre (under registration number: NL23932.029.08) and all participants gave written informed consent. The trial is registered at the Dutch Trial Register (under registration number: NTR1899).

### Procedure

After the participant had confirmed to participate and had signed the informed consent form, the baseline questionnaire was sent out. Thereafter, the concealed randomisation procedure was carried out by an independent researcher. Participants were randomly assigned to either the usual care control group (n = 159) or the intervention group (n = 181) through a stratified computerised randomisation procedure using Microsoft© Office Access 2003 software. At first, participants were stratified according to cholesterol lowering medication use, assuming that medication use implicates treatment by a general practitioner and/or medical specialist, who could have already given lifestyle advice. In addition, we expected that a decrease in LDL-C – the primary outcome of this project - because of the intervention is smaller if a participant already uses medication. Family members of the same household were clustered and subsequently randomised as a cluster to prevent contamination of the intervention effect due to spill over of communication about the intervention among participants. For this reason, the allocation ratio was 1∶1.1.

### Theoretical framework

The intervention of the PRO-FIT project was developed according to the integrated model for exploring motivational and behavioural change, the I-Change model (2.0) [21], [23]. Briefly, it assumes that the behavioural change process can be distinguished in three phases: awareness, motivation and action/behaviour. Hypothetically, due to gained knowledge and awareness of one's CVD risk, a participant will become motivated to change lifestyle behaviour(s), and subsequently, implementation intentions and action plans will be formed to actually achieve (maintenance of) behavioural change. In addition, it is assumed that this will eventually lead to a reduction in CVD risk (see figure S2).

### Intervention

The intervention consisted of a combination of tailored web-based advice (PRO-FIT*advice) and face-to-face counselling complemented with telephone booster sessions (PRO-FIT*coach). The goal was to: 1) improve awareness of the cardiovascular disease risk through an increase of specific knowledge, cues to action and change in risk perception, 2) improve motivation with respect to healthy behaviour through an increase of specific knowledge and a change in attitude, self-efficacy and social influences, 3) adopt and maintain a healthier lifestyle, with regard to physical activity, saturated fat intake, fruit and vegetables intake, smoking and compliance to statin therapy, and 4) lower the level of LDL-C and other biological CVD risk indicators and thereby reducing the CVD risk.

The intervention has been described in detail elsewhere [22]. Briefly, participants were encouraged to visit a weblink referring to the project website, where generic CVD risk information was presented, containing feedback on CVD risk behaviours, their contribution to overall CVD risk, and cues on how to change behaviours. Thereafter, participants could log on to a personal account, consisting of six tailored advice modules on smoking, physical activity, saturated fat intake, fruit intake, vegetables intake and compliance to statin therapy. The module on compliance to statin therapy was developed at the Rijksuniversiteit Groningen, the Netherlands. The other modules were based on existing tailored information modules of the ‘Healthy Life Check’ (in Dutch: ‘Gezondlevencheck’) of the Netherlands Heart Foundation [24]. The modules on fruit and vegetables were mainly based on existing modules of the Live Healthy Coach (in Dutch: Leefgezondcoach) of the Dutch Diabetes Federation, developed at the Erasmus University Medical Centre in Rotterdam, the Netherlands.

On-screen personalised feedback was tailored to personal performance level (current lifestyle behaviour), awareness of one's own performance, as well as personal motivation to change, outcome expectations, attitude and self-efficacy. Personalised feedback to compliance to statin therapy was tailored on knowledge and personal beliefs about (the effect of) statin therapy, potential side effects of the prescribed drug and current compliance.

Subsequently, the participant and the personal coach further established the level of the participant's knowledge/awareness about FH and CVD risk factors. Furthermore, the assessment(s) and advice(s) within the participant's personal PRO-FIT*advice account were discussed and ambivalence and barriers related to the recommended behaviour changes were explored based on Motivational Interviewing (MI) techniques [25]. Further, an additional one to five counsellor-initiated booster telephone sessions were performed to further encourage the participant's behavioural changes. The two personal coaches had lifestyle coaching and nursing/teaching backgrounds and had received an additional 3-day MI workshop, incorporating both introductive lessons and practical training sessions with professional actors. Both participant and personal coach were not blinded for group assignment and intervention implementation.

The control group received care as usual.

### Measurements

#### Lifestyle related outcomes

The level of physical activity in minutes of moderate to vigorous physical activity performed per week, as well as whether participants either did meet or did not meet the physical activity guideline of 30 minutes of moderate- to vigorous physical activity on at least 5 days a week [26], was measured by the Short QUestionnaire to ASsess Health-enhancing physical activity (SQUASH), which has been found to be fairly reliable and reasonably valid [27].

Saturated fat, fruit and vegetables intake were measured by the short Dutch questionnaire on total and saturated fat intake and on fruit and vegetable intake, that have been validated as related to seven day dietary records [28], [29]. For the fruit and vegetable questionnaire also biomarker validity has been established [30]. From this questionnaire, a score for saturated fat intake, ranging from 0 (lowest) to 80 (highest) fat points was computed, as well as servings of fruit and grams of vegetables per day. One fat point equals 2 gram of saturated fat. Subsequently, it was assessed whether a participant met the Dutch recommendations for daily saturated fat intake, being ≤28 gram/day for men and ≤22 gram/day for women, as well as for daily fruit intake (2 servings/day) and daily vegetable intake (200 gram/day) [31]. Smoking behaviour was assessed by a self-reported measure, asking participants if they were a current smoker, an ex-smoker, or a never smoker. Consequently, they were categorized as either smoker (if currently smoking) or non-smoker (if ex-smoker or never smoker) [32].

The five-item Medication Adherence Report Scale (MARS-5) was used to measure self-reported compliance to statin therapy, which was found to have good reliability and validity [33]. Scores on five items were combined to a total score ranging from 5 (lowest) to 25 (highest). The items referred to whether participants always (1)/never (5) forget or stop their medication, decide to miss out a dose, take less than instructed or alter the dose of their medication without consulting a medical doctor and/or pharmacist. Based on former research, low compliance is suggested if one or more doses are missing, thereby assuming an overestimation of the actual compliance [34], [35]. As a consequence, participants with a score of 25 were categorised as compliant to statin therapy, others (score<25) as non-compliant.

#### Other outcomes

Intention to change was assessed with a self-report measure, asking participants whether they plan to change behaviour X on a 5-point Likert scale (certainly yes (1) to certainly no (5)) and how sure they are of this (absolutely sure (1) to absolutely not sure(5)). Both scores were averaged and participants were categorised into motivated (average score≤2) or unmotivated (average score>2) to change behaviour for each specific behaviour [36].

Both height (in cm) and body weight (in kg) were measured twice on calibrated scales. Body Mass Index (BMI) was calculated from the average scores. LDL-C was measured with fasting finger stick samples analysed on a Cholestech LDX desktop analyser (Cholestech, Hayward, USA). The reproducibility and precision of lipids measurement by the LDX analyser are within the guidelines of the NCEP [37], [38]. The Cholestech LDX analyser has been validated for point-of-care lipid measurements in clinical practice [39].

A process evaluation was carried out, taking into account the process elements reach, dose (delivered and received) and fidelity. The research methods of this evaluation, as well as the results and discussion are extensively described elsewhere [40]. In short, reach (the number of people with FH that took part in the project, as well as how representative the participants in the intervention group were for the study population and non-participants) was assessed by consulting the StOEH client database, as well as the PRO-FIT client database. The dose of all delivered elements of the intervention was assessed by logs that were kept by the coaches and the project database. Dose received, i.e. the way participants used PRO-FIT*advice (% of participants that logged on, number of modules finished), was assessed by means of log on rates and website use data. Whether face-to-face counselling sessions were implemented as planned according to MI guidelines (i.e. MI fidelity) was assessed by two MI experts, following the Motivational Interviewing Treatment Integrity code (MITI 3.1.1.) [41].

Self-reported measures were collected through digital questionnaires that were sent by email. Body height, weight and lipid measurements were conducted at the participants' homes by a research assistant.

### Statistical analyses

Potential baseline differences were checked between intervention and control group with linear and logistic regression analyses, including group allocation as an independent variable, and the following dependent variables: gender, age, education, BMI, medication use, LDL-C and whether participants met the recommendations on the different lifestyle behaviours at baseline. In addition, differences between dropouts and non-dropouts regarding the above-mentioned baseline characteristics were tested with linear and logistic regression analyses as well. If baseline differences were found, the variable concerned was included in further analyses. Effect modification of the above-mentioned variables and intention to change was checked and confirmed if the p-value of the interaction term was <0.05. Only in case of significant effect modification, outcomes were presented per category of the effect modifier (e.g. for women and men separately) as well.

Primary, a complete case analysis was conducted at the participant level, restricted to those who filled in questionnaires at both baseline and follow-up. These numbers vary for different outcome measures. Subsequently, an intention-to-treat analysis was conducted, involving all participants who were randomly assigned (n = 340). Missing data on physical activity, dietary saturated fat, fruit and vegetable intake, smoking and compliance to statin therapy were imputed using multiple imputations. Five different datasets were created in SPSS (version 18.0) using Fully Conditional Specification and Predictive Mean Matching procedures. All available data on the above-mentioned lifestyle outcomes, as well as on group allocation, gender, age, education, BMI, medication use and LDL-C were included in the imputation model. Thereafter the multiple datasets were analysed as described below, using SPSS (version 18.0). Pooled estimates were computed following the rules as described by Rubin [42]. As no major differences were found, only the results of the complete case analysis are presented.

In order to investigate whether the PRO-FIT intervention had had an effect on physical activity, dietary saturated fat, fruit and vegetable intake, smoking and compliance to statin therapy, regression analyses were conducted. Linear regression analyses were conducted, including group allocation as an independent variable and the following continuous outcome measures as dependent variables: saturated fat intake, fruit and vegetables intake, physical activity, compliance to statin therapy). Because data on physical activity were skewed, we log-transformed them and conducted log-linear regression analyses. Binary logistic regression analyses were conducted, including group allocation as an independent variable and smoking as a dependent variable. The post-test scores were regressed on study group and baseline measure of the outcome variable.

## Results

### Baseline characteristics of participants

In Figure S1 the recruitment, participant and retention flow is presented. As can be seen from Table 1, the participants were equally distributed with regard to gender. Overall, a mainly middle-aged, medium to highly educated, fairly overweight sample participated in the project. The majority had an elevated LDL-C and used cholesterol-lowering medication. Baseline differences between control and intervention group were found for BMI (β = −1.10; CI −2.17− −0.04). As a consequence, this variable was included in the regression analyses. No differences were found between dropouts and participants regarding the baseline characteristics.

**Table 1:** Baseline characteristics of the control and intervention group.

	Control group	Intervention group
Gender (% female; N)	56.3; N = 159	57.1; N = 181
Age (years, mean ± SD; N)	45.9 (13.0); N = 159	44.7 (12.9); N = 181
Education^1^ (%; N)		
*low*	3.6	3.1
*medium*	62.8	58.2
*high*	33.6; N = 137	38.7; n = 163
BMI (kg/m^2^, mean ± SD; N )	**27.1 (5.3); N = 159**	**26.0 (4.7); N = 181**
Medication use (% yes; N)	69.6; N = 159	68.8; N = 181
LDL-C (mmol/l, mean ± SD; N )	3.7 (1.2); N = 130	3.7 (1.3); N = 146

1 Classification according to National Monitor Public Health: www.monitorgezondheid.nl.

N = sample size; SD = standard deviation; BMI = body mass index; Significant differences between control and intervention group (P<0.05) are printed in bold font.

### Effects on physical activity

No significant between-group differences were found regarding physical activity. As can be seen from Table 2, after 12 months, the control and intervention group performed more minutes of moderate to vigorous physical activity per week. The majority of both groups was compliant to the Dutch guideline of physical activity at baseline (both 78%) and after 12 months (both 80%).

**Table 2:** Lifestyle behaviours at baseline and follow-up and intervention effects from linear or logistic regression analyses.^1^

	Control group	Intervention group		
	Mean (SD);N	Mean (SD);N	β	95% CI
MVPA^2^ (min/wk)				
Baseline	363.1 (3.5); N = 146	422.0 (3.1); N = 171		
12 months	428.0 (3.7); N = 146	501.0 (3.3); N = 171		
Difference	+64.9	+79.0	1.11^3^	−0.12–0.33
Saturated fat intake (fat points/day)				
Baseline	14.3 (4.9) N = 146	15.4 (4.8) N = 171		
12 months	13.7 (4.6) N = 146	14.0 (5.0) N = 171		
Difference	−0.6	−1.4	−0.61	−1.35–0.14
Fruit intake (servings/day)				
Baseline	1.4 (1.1); N = 145	1.5 (1.3); N = 169		
12 months	1.4 (1.1); N = 145	1.6 (1.1); N = 169		
Difference	+0.0	+0.1	0.05	−0.12–0.22
Vegetables intake (grams/day)				
Baseline	151.2 (77.8); N = 144	162.1 (75.8); N = 169		
12 months	163.4 (77.2); N = 146	171.5 (76.6); N = 169		
Difference	+12.2	+9.4	3.26	−9.78–16.29
Smokers (%)				
Baseline	15.2; N = 145	18.3; N = 171		
12 months	10.2; N = 146	13.5; N-171		
Difference	−5	−4.8	OR = 1.15	0.39–3.33
Compliant to statin therapy (%)^4^				
Baseline	44.4; N = 99	38.1; N = 118		
12 months	51.4; N = 105	44.5; N = 119		
Difference	+7.0	+6.4	OR = 0.99	0.51–1.94
Post-hoc analyses				
Saturated fat intake (fat points/day) **in men**				
Baseline	16.3 (5.3); N = 63	16.7 (4.9); N = 73		
12 months	15.2 (4.5); N = 63	15.5 (5.2); N = 73		
Difference	−1.1	−1.2	−0.06	−1.30–1.16
Saturated fat intake (fat points/day) **in women**				
Baseline	12.8 (3.9); N = 82	14.4 (4.5); N = 98		
12 months	12.6 (4.4); N = 83	12.8 (4.6); N = 98		
Difference	−0.2	−1.6	**−1.03**	**−1.98–−0.08**

1 Differences between control and intervention group after 12 months are tested through linear of logistic regression analyses, controlled for baseline values and baseline BMI. N = sample size; SD = standard deviation; β/OR = beta or Odds ratio as effect indicators from linear or logistic regression analyses; 95% CI = 95% confidence interval as effect indicator from linear or logistic regression analyses; Significant differences between control and intervention group (P<0.05) printed in bold font.

2 MVPA = Physical activity with moderate to vigorous intensity; means are geometric means;

3 Log-linear regression was conducted;

4 Assessed with the MARS-5 questionnaire, a score = 25 is defined as compliant, < = 24 is defined as noncompliant.

Since no major differences were found between intention-to-treat analysis and complete case analysis, only the results of the complete case analysis are presented.

### Effects on saturated fat and fruit and vegetable intake

After 12 months, the control and intervention group consumed less fat points compared to baseline values. No significant between-group effect was found. Gender appeared to be a significant effect modifier (p = 0.03). Post-hoc analysis showed a significant decreased fat consumption specifically among women in the intervention group compared to the control group after 12 months (see Table 2). In general, after 12 months, 13% more participants in the intervention group met the recommendations for fat intake, compared to 1% more in the control group.

No significant between-group differences were found regarding fruit intake. A minimal change was seen in the amount of servings of fruit per day consumed by both control and intervention group after 12 months (see Table 2). In both control and intervention group, the percentage of participants meeting the recommendations for fruit intake slightly increased (+2% and 7%).

No significant between-group differences were found regarding vegetables intake. More grams of vegetables per day were consumed in both control and intervention group after 12 months (see Table 2). After 12 months, 12% more participants in the control group met the recommendations for vegetable intake, as opposed to 4% more participants in the intervention group.

### Effects on smoking behaviour

No significant between-group effect was found on smoking behaviour. A decrease in the overall percentage of smokers was seen in both control and intervention group after 12 months (see Table 2). Changes in smoking behaviour were similar in both groups. The majority (control group: 80%; intervention group: 85%) continued not-smoking, and 13% (control group) and 10% (intervention group) continued to be a smoker. Respectively 7% (control group) and 5% (intervention group) quitted smoking in the past year, and 1% in both groups started smoking.

### Effects on compliance to statin therapy

No significant between-group effect was found on compliance to statin therapy. Of the participants who used cholesterol lowering medication at baseline, 44% of the participants in the control group was categorised as compliant at baseline, associated with a score of 25 on the MARS-5 questionnaire, compared to 38% in the intervention group. After 12 months, an increase in compliance was seen in both the control group and the intervention group.

### Process

A 34% (n = 181) representative proportion of the intended intervention group was reached during the recruitment phase; participants did not differ from non-participants (n = 623) on age, gender and LDL-C levels. Of the participants, 95% received a PRO-FIT*advice log on account, of which 49% actually logged on and completed at least one advice module. Nearly all participants received a face-to-face counselling session and on average, 4.2 telephone booster calls were delivered. None of the face-to-face sessions were implemented according to MI guidelines.

## Discussion

In this paper, we aimed to investigate the efficacy of an individualised lifestyle intervention on physical activity, dietary intake, smoking and compliance to statin therapy among people with FH. After 12 months, improvements were seen in both control and intervention group in physical activity, saturated fat intake, fruit and vegetable intake, smoking and compliance to statin therapy. Although most changes were more pronounced among participants in the intervention group, the between-group differences were small and not significant. Post-hoc analyses showed a significant decrease in the intervention group in saturated fat intake among women.

This lack of effects is in contrast with the latest evidence in the field of computer-tailored promotion of healthy lifestyle behaviours; recent reviews and meta-analyses indicate that such tailored interventions are likely to be effective [18], [25], [43]–[47] [48]–[50]. However, evidence on the effects of such and other lifestyle interventions in a FH population is scarce. In a review on dietary interventions in a FH population, Shafiq and colleagues emphasise the need for large, parallel randomised controlled trials, since no reliable conclusions could be drawn from the included studies [51]. Until now, no indisputable effects have been published so far.

It may be that the intervention reach and true exposure (dose received) was insufficient to initiate behaviour changes. The content of the intervention was largely based on earlier tailored interventions, that were effective on behaviour changes, and our process evaluation indicates that participants were sufficiently exposed to the intervention. However, the results also indicate that only half of the participants logged on at the PRO-FIT*advice website and completed at least one of the advice modules, and that face-to-face counselling sessions were delivered with low MI fidelity. Mixed evidence has been published on computer-tailored interventions addressing more than one lifestyle behaviour. In their latest review, Sweet and colleagues concluded that single health behaviour interventions are more effective at changing specific health behaviours than multiple-behaviour interventions [52]. Further, it appears from literature that multiple-behaviour interventions may be burdensome for some individuals, and advices may be too long [53]–[55]. Regarding the low MI fidelity, it has often been reported that skills required for effective MI may take longer to develop than the 3-day MI workshop in our project [56], [57]. Probably, the provided MI workshop was not sufficient and more thorough monitoring and supervision of counselling skills during the intervention should have been built in.

The lack of large improvements in both control and intervention group, might be caused by the relatively healthy lifestyle of our population. Results showed that the majority of the people with FH in this project already met the recommendations on physical activity and smoking behaviour at baseline (physical activity: 78%; non-smokers: 81–85%). However, this was the case for both control and intervention group. On this point, the FH population obviously differed from the general Dutch population, as survey data show that only 53% of the Dutch general population is sufficiently physically active and 73% of all Dutch adults are non-smokers [58] [26]. Though, there was much room for improvement with regard to saturated fat and fruit and vegetable consumption. Only 49–57% of our study population met the Dutch recommendations on saturated fat consumption, and only one third on fruit and vegetable consumption.

The baseline self-reported compliance to statin therapy in our project (38–44%) is comparable to those reported in the literature. Our results showed no significant intervention effect. According to recent reviews, the effects of compliance-improving interventions are generally small [34], [59]. About 50% of the interventions proved to be efficacious, and effects on treatment outcomes (p.e. LDL-C) were often absent. So far, little is known about the determinants of compliance [34]. Julius and colleagues recommended assessing patients' motivation to take prescribed medications, and to identify and address potential barriers to compliance [60].

To our knowledge, the PRO-FIT intervention is the first to evaluate the effects of an innovative lifestyle intervention on multiple lifestyle behaviours among people with FH. The RCT was conducted in a sample representative for the general FH population with a small drop-out rate. The intervention is innovative in combining three communication channels: the individualised web-based approach added by the social interaction of the face-to-face and telephone coaching sessions. So far, few studies have evaluated the effects of an intervention that had combined web-based computer-tailored lifestyle education *and* motivational interviewing techniques on multiple lifestyles [61]–[63]. Thereby, the step-wise approach of raising awareness first, then giving tailored feedback and thereafter motivating people towards behavioural change, is thoroughly described and based on a firm theoretical framework [22], [23]. Moreover, from the process measures reach and dose it can be said that the implementation of the intervention was feasible. Confidence in the validity of our findings is increased by the parallel randomised study design and absence of differential attrition.

This project also had limitations. Behaviour is multi-dimensional and complex to measure by self-report. The use of inappropriate or crude measures has serious implications and could likely have led to misleading results, for instance an underestimation of effect sizes. Although fairly reliable and valid questionnaires were used, the choice of a (self-report) measure often remains a compromise between the research aim, accuracy level and feasibility [64].

Despite randomization of 4 clusters of family members living in the same household, communication among family members of control and intervention group was unavoidable. The Dutch screening program works cascade-wise; once a person is diagnosed (the index patient), pedigrees are consulted to trace other potentially FH positive family members. In a relative small country such as the Netherlands, families appeared to be wide-spread and overlapping each other, making it rather challenging to prevent communication, which therefore should be taken into account when interpreting the results.

## Conclusions

In conclusion, this project suggests that in general individually tailored feedback is not superior to generic feedback regarding changes in multiple lifestyle behaviours in people with FH. Women aged 18–40 years in the intervention group consumed significantly less saturated fats, and compliance to statin therapy significantly improved among unmotivated medication users in the intervention group. These results should be carefully interpreted, due to post-hoc analyses of relatively small subgroups. Research is needed to gain more insight in the characteristics of this specific high-risk population, for instance risk perceptions and determinants of behaviour, such as self-efficacy, attitude, motivation and social influence. The effects of the small lifestyle changes on CVD risk remains (and is due) to be investigated.

In practice, it is crucial to achieve an optimal received dose of a computer-tailored intervention, by e.g. reducing the burden of filling in (screening) questionnaires to a minimum in order to keep participants motivated, e.g. by creating a joint questionnaire, for both evaluative and tailoring purposes. Thereby, it is known that incorporating iterative feedback and interactive website components are positively associated with exposure to web-based interventions [65]. Further, MI training of counsellors should be more extensive, incorporating more thorough monitoring and supervision of counselling skills.

## Supporting Information

Figure S1 **Recruitment, participant and retention flow.** People diagnosed with FH from January 1^st^ 2007 to April 15^th^ 2009, aged from 18 to 70 years, with a LDL-C level>75^th^ percentile (age and gender specific), with access to internet, sufficient fluency in Dutch and residency <150 km radius from Amsterdam were considered as eligible for participation and recruited from the national cascade screening programme of the Foundation for the Identification of Persons with Inherited Hypercholesterolemia (StOEH). Invitation brochures were send to 986 people. The recruitment period lasted 6 months and resulted in 340 participants. Three hundred and eighteen participants (94%) completed the baseline and follow-up questionnaires. Missing data on physical activity, saturated fat intake, fruit and vegetables intake, smoking and compliance to statin therapy were imputed using multiple imputations, allowing an intention-to-treat analysis based on 340 participants.
(TIF)

Figure S2 **The I-Change model 2.0.** The I-Change model assumes that the behavioural change process can be distinguished in three phases: 1) Awareness, 2) Motivation and 3) Action. Hypothetically, due to gained knowledge and awareness of one's CVD risk, a participant will become motivated to change lifestyle behaviour(s), and subsequently, implementation intentions and action plans will be formed to actually achieve (maintenance of) behavioural change. In addition, it is assumed that this will eventually lead to a reduction in CVD risk.
(TIF)

Protocol S1 **Trial protocol.**
(PDF)

Checklist S1 **CONSORT checklist.**
(DOC)
