# Supplementary figures and images for: Can Multiple Lifestyle Behaviours Be Improved in People with Familial Hypercholesterolemia? Results of a Parallel Randomised Controlled Trial
Source: PLoS One. 2012 Dec 12;7(12):e50032. doi: 10.1371/journal.pone.0050032 (PMC3520968; doi:10.1371/journal.pone.0050032)

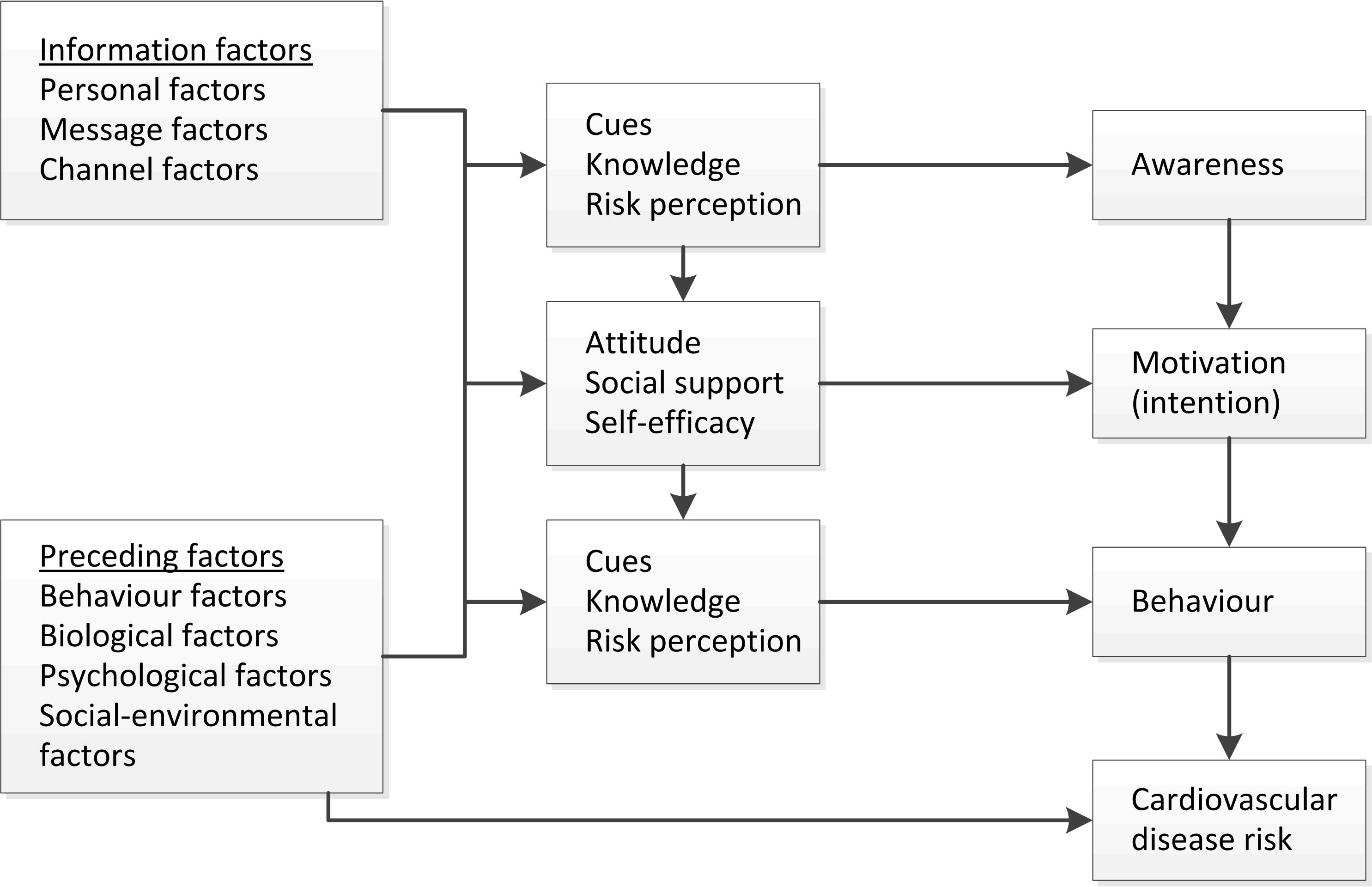

Supplement: Figure S2 — The I-Change model 2.0. The I-Change model assumes that the behavioural change process can be distinguished in three phases: 1) Awareness, 2) Motivation and 3) Action. Hypothetically, due to gained knowledge and awareness of one's CVD risk, a participant will become motivated to change lifestyle behaviour(s), and subsequently, implementation intentions and action plans will be formed to actually achieve (maintenance of) behavioural change. In addition, it is assumed that this will eventually lead to a reduction in CVD risk. (TIF) [file pone.0050032.s002.tif]
